# Data-driven meal events detection using blood glucose response patterns

**DOI:** 10.1186/s12911-023-02380-4

**Published:** 2023-12-08

**Authors:** Danilo F. de Carvalho, Uzay Kaymak, Pieter Van Gorp, Natal van Riel

**Affiliations:** 1 Jheronimus Academy of Data Science, Eindhoven University of Technology, ‘s-Hertogenbosch, The Netherlands; 2 Industrial Engineering & Innovation Sciences, Eindhoven University of Technology, Eindhoven, The Netherlands; 3 Department of Biomedical Engineering, Eindhoven University of Technology, Eindhoven, The Netherlands

**Keywords:** Meal detection, Continuous glucose monitoring data, Real diabetes data, Pattern identification, Distance profile

## Abstract

**Background:**

In the Diabetes domain, events such as meals and exercises play an important role in the disease management. For that, many studies focus on automatic meal detection, specially as part of the so-called artificial $\beta$-cell systems. Meals are associated to blood glucose (BG) variations, however such variations are not peculiar to meals, it mostly comes as a combination of external factors. Thus, general approaches such as the ones focused on glucose signal rate of change are not enough to detect personalized influence of such factors. By using a data-driven individualized approach for meal detection, our method is able to fit real data, detecting personalized meal responses even when such external factors are implicitly present.

**Methods:**

The method is split into model training and selection. In the training phase, we start observing meal responses for each individual, and identifying personalized patterns. Occurrences of such patterns are searched over the BG signal, evaluating the similarity of each pattern to each possible signal subsequence. The most similar occurrences are then selected as possible meal event candidates. For that, we include steps for excluding less relevant neighbors per pattern, and grouping close occurrences in time globally. Each candidate is represented by a set of time and response signal related qualitative variables. These variables are used as input features for different binary classifiers in order to learn to classify a candidate as Meal or Non-Meal. In the model selection phase, we compare all trained classifiers to select the one that performs better with the data of each individual.

**Results:**

The results show that the method is able to detect daily meals, providing a result with a balanced proportion between detected meals and false alarms. The analysis on multiple patients indicate that the approach achieves good outcomes when there is enough reliable training data, as this is reflected on the testing results.

**Conclusions:**

The approach aims at personalizing the meal detection task by relying solely on data. The premise is that a model trained with data that contains the implicit influence of external factors is able to recognize the nuances of the individual that generated the data. Besides, the approach can also be used to improve data quality by detecting meals, opening opportunities to possible applications such as detecting and reminding users of missing or wrongly informed meal events.

**Supplementary Information:**

The online version contains supplementary material available at 10.1186/s12911-023-02380-4.

## Introduction

Every day, millions are impacted globally by the chronic disease of diabetes [1]. Such condition is heavily associated with the control of the amount of glucose in the blood that should remain within a particular range. This implies an intrinsic type of self-management in the life of a person with diabetes, where managing daily events associated to food intake, exercises, and insulin plays a critical role.

To mitigate the disease burden, researchers develop solutions such as personalized medication recommender systems or automatic insulin pumps. For that, data gathered from patients are crucial. Data collection becomes then a core step in the development of such solutions, and fortunately a major part of patients with diabetes is willing to provide input to researchers. This step can be supported by the use of wearables such as smartwatches, that can collect self-logged data through self-reports (e.g., registration of meals, mood, insulin shots, medication), or passively through sensors (e.g., collecting heart rate, displacement, steps). Such gathered data can serve as the basis for data-driven approaches [2–5]. However, the fact of adding a new “task” to their routines – even if for the sake of research – also poses an additional burden on patients, especially for long-running studies [6].

Several factors can be taken into account when dealing with blood glucose (BG) variation, and a very common and important one is meal information [7]. This means that information around food intake is valuable, in particular accurate information on the timing of a meal event, which brings to the table a very error-prone scenario: during their day, patients must constantly inform through a device when they had a meal. Unfortunately, for the solutions that depend on it, self-reported data on meal or snack intake will not come without errors and uncertainty [8]. While pure sensor-based approaches also have their limitations, it is promising to combine sensor data with self-reported data to correct or supplement erroneous or forgotten self-reports automatically.

### The challenge and value in meal detection

Diabetes management systems rely on detecting BG variations (e.g., meal events detection). For instance, in artificial pancreas systems, their insulin pump control is able to manage the insulin injections properly and automatically [9–13]. Such control systems are idealized and developed around what is called “The meal challenge”, which in summary can be tackled according to three scenarios involving meal events as input:

1. **Feed-forward control**: each meal is self-reported to the control system by the user at the moment it occurs (or is about to occur).
2. **Feed-back control**: by keeping track of CGM data collected by a sensor, the control system responds to every large rise in glucose. This, however, has proven difficult in practice due to the trade-off between a needed quick response (to rapidly cope with the insulin absorption delay) and a possible insulin overdose.
3. **Discrete meal detection**: through a continuous feedback – also keeping track of CGM data – a specialized algorithm triggers insulin injection when a meal event is detected.

Each of the scenarios could be implemented/used independently, however a combination of **1.** and **3.**, or **2.** and **3.** could definitely exist.

It is clear that meals are a key event tied to BG value variation, and so inferring them becomes a valuable task. Meal detection approaches focusing on glucose rate of change do exist, however their strict focus on blood glucose information lead them to error, owing first to noise in the signal, and secondly to events such as physical activities (which can increase BG) that overlap with meal events [9, 10]. Thus, as factors such as stress and physical activities also affect BG and insulin sensitivity, the so-called “Meal challenge” is now expanded, turning into an even more complex problem that includes multiple variables interfering in the BG signal.

### The proposed approach

To be able to include external factors and information, a data-driven approach comes as a natural solution, as different types and sources of data can be put together, and they all can contribute to the solution. The premise here is that a model able to work with a set of information/features could also work with an expanded and more complete version of this same set. As new data and features come in, the developed model could turn into a potential better and more specialized version of itself. This is true for a model created from individual level generated data (in our case, per individual/person), or from population level data (from a set of individuals/people), which opens opportunities to the development of both personalized and general models [14]. While, in theory, more features may benefit model performance, this also calls for more streams of input data with additional risks for missing and erroneous registrations. The models created must therefore cope with low data availability and quality, and implicit uncertainty in self-logged data.

This paper focuses on evolving the idea of tackling the meal detection challenge through recurring meal response patterns found in the data. This allows for a model with an adaptive behavior: it is able to identify specific (personal) types of change that must be interpreted as meal responses strictly from data, instead of considering pre-defined rules and change values to be applied to all signals/data. When put in the perspective of free living daily events, response patterns found in the glucose signal can be used to detect new occurrences of such continuous subsequences [15]. However, finding multiple occurrences and distinguishing among them which one should be taken as a proper meal event is still left as an open problem. The amount of false positives tend to grow when more meal candidates are considered (e.g., different pattern matches in a day), and thus the issue remains on how to find the best fitting candidate among the matches.

The paper is presented as follows. In Related work section, we present related research and their relevant limitations. Details of the proposed method are presented in Methods section, together with the associated concepts. Experimental setup and Results are presented in the following two sections. Finally, Conclusions are given and discussed.

## Related work

Studies on mitigating imperfections in the collected data were done previously [14, 16, 17] with the aim to infer missing events – including meals – and impute them to create a better version of the data. In these works, the inference is made by calculating the likelihood of having an event (activity) within a chained sequence of informed events. For that reason, previous and/or future event information must be taken as input when training imputation models.

Approaches for detecting meals though BG variation rely on figuring out specific changes in the incoming BG levels to detect the events. Relying solely on the BG signal as input, such approaches would allow for less to no patient-device interactions, as such input can be passively acquired by a continuous glucose monitoring (CGM) sensor. This also opens opportunities for such solutions to be used on improving artificial pancreas applications and diabetes related simulators [10, 18, 19], also enabling “precision nutrition”.

From a data-driven perspective, the meal detection challenge can be tackled using different routes [13]:

- **Analysis of event orders:** Events are seen as a sequence of states that tend to happen in specific orders.
- **Analysis of glucose variation:** Change points can be detected using pre-determined BG rate of change thresholds, flagging a meal.
- **Analysis of glucose signal patterns:** Chunks of the BG signal that very often appear after meals can be identified as response patterns.

If isolated from the BG signal, and seen as a sequence of ordered activities, the meal occurrences can be seen and dealt with as a chain of states with probability values associated to the transition between such states, and this be used for predicting the occurrence of a (meal) activity [14, 20–23]. Focusing on the BG signal, a value computed and associated to the glucose level rate of change can be seen as a trigger of a glycemic response to a meal [9, 24], and by making use of a filter, a qualitative representation of this same signal can be the input for a carbohydrate estimation algorithm [25, 26]. In the same manner, BG signal’s first and second derivatives can be tied to pre-defined (heuristic) rules able to detect unannounced meals in a margin of 30 to 60 minutes after the event [27].

The previous approaches share a trend in making the meal detection based on glucose variation using predetermined BG rate of change thresholds. They apply such rules to all data without considering any individual distinction. Furthermore, there is no intrinsic consideration of external factors that can only be seen in patterns retrieved directly from the data itself.

## Methods

Our methods aim at using identified BG response patterns to spot similar occurrences in signals coming from real CGM data, and classify them as meals. These response patterns originate from daily-living self-reported meal events, such as breakfast, lunch, dinner, snacks, and hypo-correction (e.g., sugary drinks like juice or regular soda). For that, a data set containing CGM data together with the associated recorded meal events from multiple users (participants) is used as input, more specifically the *OhioT1DM* [28] data set (to be detailed in Experimental setup section).

### Data description

**Fig. 1:**
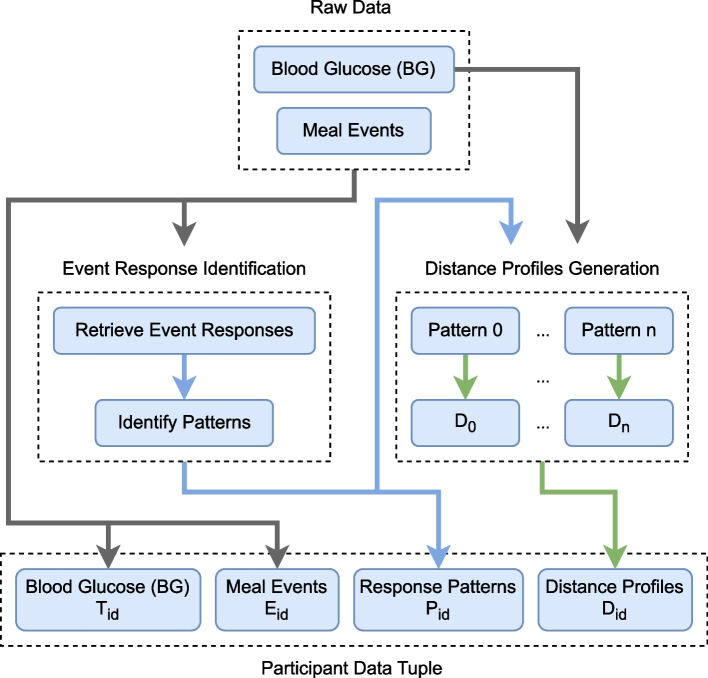
Diagram of the participant data tuple. The diagram presents the pieces of data that form a participant data tuple used as input for the methods

Figure 1 summarizes the data used per participant as input for the methods presented in the proposed work.

Assuming individual (per participant) data is available to the study, and each participant is associated to a unique identifier (*id*), each participant dataset $X_{id}$ can be denoted by the tuple:

1 $$\begin{aligned} X_{id} = \left( T_{id}, E_{id}, P_{id}, D_{id}\right) \end{aligned}$$

where $T_{id}$ and $E_{id}$ are time series of BG and meal events, respectively, sharing the same time space, $P_{id}$ 1 the set of response patterns found, and $D_{id}$ a set containing the distance profiles [29] generated using each of the identified patterns as queries applied to $T_{id}$. For short, $X_{id}$ can be referenced as $X = (T, E, P, D)$, as the following sections cover methods always applied on a participant level data, and able to be used on any participant, allowing *id* to be seen as an implicit/hidden variable. The sections to follow cover all elements of the participant data tuple, explaining how they fit the methodology, and also the concepts behind them.

### Event responses

In a continuous BG signal over time (CGM), responses to events that trigger variations can be seen as changes in the flow of such signal. Making use of both CGM and logged meal events data, it is possible to pinpoint in the BG signal when each known logged meal happened, considering both data (time series) were acquired altogether. The continuous chunk of the BG time series that comes after a logged meal can be seen as a response to that meal.

#### Definition 1

(Meal Response) Given a BG time series $T = t_1, t_2, \dots , t_m$, and a logged meal events time series $E = e_1, e_2, \dots , e_k$, a response $T_{i,n}$, to a given logged meal event, $e_k$, is a continuous subset of length $n \le m$ composed by contiguous positions from *T*, that is a subsequence $T_{i, n} = t_i, t_{i+1}, \dots , t_{i+n-1}$, where $1 \le i \le m-n+1$, and *i* is the data point associated to the logged meal event $e_k$.

It is worth mentioning that in our study, the response size is a pre-defined time interval $\Delta _{response}$, which means that while varying in form, every subsequence $T_{i,n}$ taken as a response to a meal event $e_k$ gathered from the data has the same length. In particular, in our data analysis, we opted to rely on such easy to adjust constant that ensures that CGM responses within a period after a meal are used to train (and later apply) a model for automatic meal identification.

### Response patterns and candidates selection

Taking as basis the work done in [15], for a set of meal responses taken from a given *E* and *T*, a set of response patterns $P=\{p_0,p_1,\dots ,p_i\}$ is identified, as well as the associated distance profiles $D=\{d_1,d_2,\dots ,d_i\}$ in regard to *T*, being *D* the core of the pattern occurrence search – in other words, the selection of matches.

It is fair to assume that the task of selecting matches has a critical role in the method, and also that there is an implicit issue regarding the top-*n* approach used in [15]. Aiming at attenuating the previous top-*n* approach’s limitation, an alternative selection procedure was developed: the selection of candidates, incorporated into the proposed method as a direct improvement over the former. The new procedure selects a dynamic number of matches to the patterns (in opposition to the top-*n* selection) while filtering them, creating this way better suitable subsequences to be taken as meal responses, i.e., better candidates for classification. The proposed selection approach relies on the use of two new introduced parameters: $d_{cutoff}$, a filtering threshold that avoids selecting candidate subsequences with low similarity to the identified patterns; and $\Delta _{valley}$, which controls the distance kept over neighbor selected candidate subsequences to maintain diversity over time.

The details for the steps here covered, from the identification of the response patterns to the selection of candidates, are given in Additional file 1. It includes details covering both selection procedures, presenting their respective algorithms in sections Looking for Matches (Algorithm 1) and Candidates Selection (Algorithm 2).

### Candidates classification

The BG signal can also be seen as a set of meal responses: each data point marks the start of a subsequence that can represent a potential response to a meal. Nonetheless, a potential meal response would exist for every single data point in the signal, which would lead to an extensive search space of possible responses to be analyzed.

When translating the BG signal into binary classified response entries (Meal or Non-meal) to be used as input for training, the amount of negatives would exceed the number of positives in a heavily unbalanced proportion, which would make the task of training the classifiers more difficult [30]. Hence, reducing such search space is needed to allow for a better positives $\times$ negatives balance. For that, the already detailed selection of candidates come as a filtering/selection tool. All meal responses within the BG signal are taken, and from it, only the selected candidates are used as training data for binary classifiers [13]. Then, the trained classifiers will be able to tell if a new incoming candidate must be classified as Meal or Non-meal.

#### Preprocessing pipeline

To supply data for the training step of the classifiers, specific transformations applied to the training data are made to translate it into a set of meal response candidates. This is done by using as input the participant data tuple defined in Data description section of Methods. For an existing participant data tuple $X = (T, E, P, D)$, the pipeline for the *training* set can be defined as:

1. Gather the BG (5-minute frequency) signal, *T*, including existing gaps.
2. Synchronize the logged meal events *E* with the BG signal, by associating the logged meal time stamps to the nearest BG data points.
3. Extract meal response subsequences, and identify meal response patterns *P*.
4. Generate the distance profiles *D* for each of the identified patterns in *P*.
5. Select candidates using the distance profiles in *D*.
  Exclude non-contiguous candidates, i.e., candidates defining responses that contain one or more gaps.
6. For each selected candidate, store extra information associated to their response.

Each candidate instance contains a set of qualitative variables that are formed by features extracted from their response subsequence. The premise is that such qualitative representation may be more relevant for detecting significant differences and changes [31–35] among the set of candidates. The types of extracted information associated to each candidate can be seen in Table 1.

**Table 1:** Classification features. Features associated to a candidate and used by the classifiers to identify a Meal

Feature	Description	Unit
Blood Glucose (BG)	BG value from CGM	mg/dl
BG derivative	Estimated derivative of the BG value	mg/dl/min
Elapsed Hours	Amount of hours passed since 00:00 of the data point’s timestamp day	hours
Response Min	Minimum BG value within the subsequence starting from the data point	mg/dl
Response Minutes to Min	Number of minutes needed to go from the start of the subsequence to its minimum value	minutes
Response Derivative to Min	BG/minutes rate from the start of the subsequence to its minimum value	
Response Max	Maximum BG value within the subsequence starting from the data point	mg/dl
Response Minutes to Max	Number of minutes needed to go from the start of the subsequence to its maximum value	minutes
Response Derivative to Max	BG/minutes rate from the start of the subsequence to its maximum value	
Meal^a^	Flag set if response is associated with an existing logged meal	{0,1}

^a^This flag represents the binary Meal, Non-Meal classes, and only exists in candidates used for the training phase

It is important to mention that for a candidate to be flagged as a Meal, we consider how distant it is to the closest logged meal event, i.e., if an event was reported as happening at time *t*, and $\delta _{margin}$ is the used margin value, the start of the candidate’s response must be placed within the interval $[t - \delta _{margin}, t +\delta _{margin}]$. For instance, with $\delta _{margin}=1$ hour, candidates are marked as Meal if placed within 1 hour before or after a self-reported meal, and as Non-Meal otherwise.

It is worth noting that, while the focus of the presented work is on diabetic data, the features extracted from the BG responses are not peculiar to this domain, in fact this same types of features could be extracted from signal responses coming from data of other domains, and applied in the same manner to similar problems, e.g., instead of BG responses to meal events, heart rate responses extracted from PPG sensor signals to identify exercise events or emotion variations.

#### Classification model selection

By using the preprocessed data, a set of binary classifiers [36] are trained and compared (*N.B.* per participant). This type of classifier fit well the problem on hands, as each trained model must be able to discern on which candidate must be classified as Meal or Non-Meal. In addition, these instances of classifiers allow for ensemble methods, i.e., more complex classifiers made out of a combination of other binary estimators [37], leaving room for better adaptation/optimization for the problem, data, and domain where the method will be applied. The comparison is made over the results of the evaluated metrics applied to each participant’s *validation* set.

## Experimental setup

### Candidates selection parameters

The proposed method requires a set of parameters, and Table 2 contains all values used for the method application. In summary:

- When analyzing BG responses in CGM data, meals tend to interfere to the signal with some delay [9, 10] due to overlapping external factors (e.g., exercises, and insulin). In our data-driven strategy, such interferences are not given explicitly as inputs to the model, they are considered implicit to the response signal, and to cover that a value of $\Delta _{response}=2$ hours was taken as a reasonable response size, a value able to keep the approach on par with previous ones [18, 26, 27, 38, 39].
- The fact that participants could have logged their entries in the beginning of their meals, during, or after adds more uncertainty to the top of the aforementioned BG response delay. This results in an implicit error regarding the timestamp of the self-logged meal event. To take this into account, $\delta _{margin}=1$ hour was used: a candidate is considered a positive if it is placed within $\delta _{margin}$ from a reported meal.
- The study performed in [15] shows that 3 response patterns are able to capture enough relevant occurrences regarding the BG signals of the *OhioT1DM* participants, which led to the same choice in the work here proposed ($|P|=3$).

**Table 2:** Parameters set for candidates selection method. Description and values of the parameters used when applying the proposed candidates selection method

Description	Parameter	Value
Response size	$\Delta _{response}$	2 hours
Success margin	$\delta _{margin}$	1 hour
Number of patterns	|*P*|	3
Distance cutoff	$d_{cutoff}$	4
Valley size	$\Delta _{valley}$	2 hours

### Binary classifiers

For the classification task within the proposed method, binary classifier types were chosen. The main metrics used for such choice were that each classifier should (i) be well known out-of-the-box methods, (ii) allow for reaching model instances of relatively usable state requiring minimal data preparation, and optimization, and finally (iii) provide ensemble capabilities [36, 40–44]. The types selected are listed and briefly described as follows:

AdaBoost: Use majority vote of a set of “week” estimators applied to modified versions of the input data.
Decision Tree: Based on simple decision rules, a tree is created, able to compare values of a set of features leading to a certain decision.
Gradient Boosting: Additive model, aiming at optimizing a loss function by consecutively fitting new models (base-learners) to be maximally correlated with minimization of the loss values.
Gaussian Naive Bayes: Simple predictive model based on conditional independence between pairs of features.
MLP: Multi-layer Perceptron is a basic form of a neural network, commonly used as a model for non-linear classification problems.
RandomForest: Uses a random portion of the features to generate decoupled decision trees instances used for voting, thus avoiding overfitting.

The application of a fundamental models such as these can be taken as waypoints to more complex models that derive from the same concepts. For instance, complex neural network models that – due to sharing concepts and structure – could be a direct and natural choice over MLP.

All binary classifiers used were instantiated using their most standard set of parameters, due to the fact that in our method, each classifier serves only as a tool that could be easily replaced in the pipeline. The main goal was to experiment with different types in order to validate the application of binary classification in our transformed participant data tuples.

As part of the implementations done in our experiments, Scikit-learn^2^ and stumpy^3^ packages [45, 46] were used, both open source projects related to machine learning (for classifiers) and distance profile (for MASS) implementations, respectively.

### Used dataset and preprocessed data

Briefly introduced in Methods section, the dataset used in our experiments is the *OhioT1DM*. This is a dataset made publicly available^4^ to facilitate research involving diabetic data, and more specifically blood glucose level prediction modeling [5]. It contains data from continuous glucose monitoring (measured every 5 minutes) of 12 participants (Type-I diabetic patients), as well as daily self-reported events. As the focus of this paper is on meal events, together with the BG signal, we retrieved from the dataset events such as breakfast, lunch, dinner, snacks, and hypo-correction. For the modeling phase, *training* and *testing* subsets already defined by the *OhioT1DM* authors were respected. Keeping the splitting as standard as possible is a general request by the same authors as a way to allow replicability of methods and further unbiased comparison of developed models based on such dataset. However, due to our model selection step, a *validation* subset is needed, and for that the last part of the *training* subset was used. The *validation* subset has the same size as *testing*, resulting in a *training* - *validation* - *testing* splitting of approximately $50\%$ - $25\%$ - $25\%$.

**Fig. 2:**
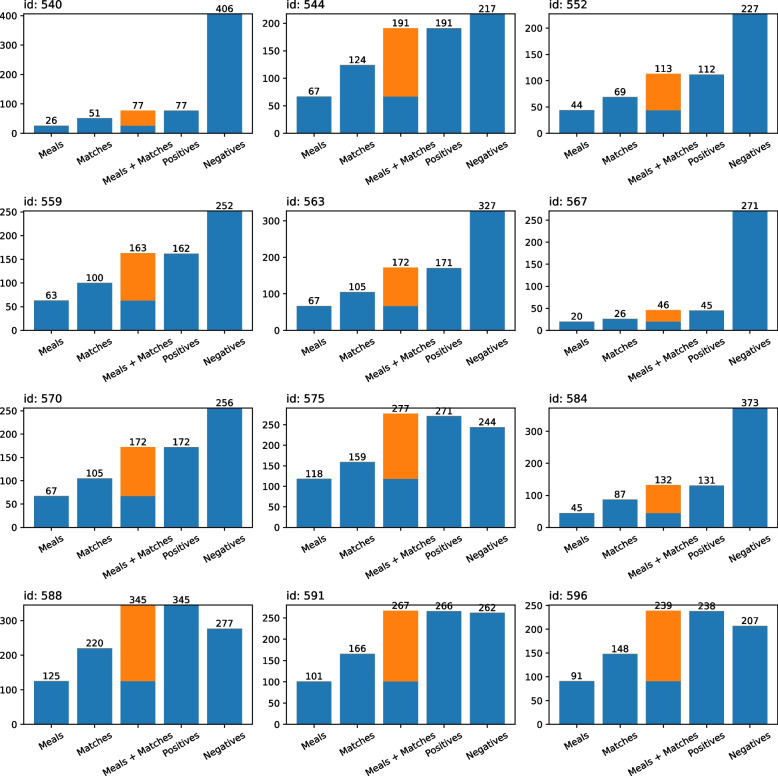
Data points, positives, and negatives. Number of data points in the preprocessed *training* set, positives (candidates classified as Meal), and negatives (candidates classified as Non-Meal) entries

The data is transformed before being used as input to the proposed method. This is done by applying the preprocess pipeline, described in Preprocessing pipeline section of Methods section, on each of the participants’ data tuples. Figure 2 shows the entries used during the training phase.

There is an important peculiarity regarding the preprocessed training data: the set of positives is composed by not only the Meal candidates, but by the logged meals that were matched by them as well. This is done as a form of maintaining the ground truth intact when training the classifiers. However, note that, for some participants, the number of positives is different from the sum of meals and matches. This happens since, due to the nature of the selection procedure, more specifically to the similarity threshold with $d_{cutoff}$, it might happen that there are regions in the BG time series where no candidates are placed around any meals. These types of dangling reported meals – without matching candidates – are not included in the *training* set, as the classifiers are supposed to be trained for candidates classification, and these types of meal events do not support any candidates.

## Results

By making use of the data described in the previous section, the outcomes of the application of the method steps are explored in this section.

### Training and validation: classifiers

Six different classifiers were trained in order to differ candidates that must be seen as a meal, and the ones that must not. *F*$_\beta$*-score*, a well known performance measure for binary classifiers [47, 48], was taken as the selection metric with $\beta =\{1, 2\}$, and the *F*$_2$*-score* used to identify the one to be used. This specific accuracy related metric measures the balance between the *precision* (PPV) and *recall* (TPR) of the test, and ranges from 0 to 1, where 1 indicates perfect precision and recall. The $\beta$ value indicates the degree of importance of the *recall* over the *precision*. Table 3 presents a summary of the evaluation results for the classifiers trained and validated for each of the participants.

**Table 3:** Model Selection metrics for all participants. Resulting values of the model selection metrics of the models validated on the data of each of the participants

ID	Classifier	Meals	Predictions	FP	FN	TP	PPV	TPR	F$_1$-score	F$_2$-score
540	Decision Tree	23	9	4	18	5	0.56	0.22	0.31	0.25
544	Random Forest	31	43	15	3	28	0.65	0.90	0.76	0.84
552	MLP	3	14	13	2	1	0.07	0.33	0.12	0.19
559	Gaussian NB	25	32	18	11	14	0.44	0.56	0.49	0.53
563	Decision Tree	25	34	19	10	15	0.44	0.60	0.51	0.56
567	Gradient Boosting	1	6	5	0	1	0.17	1.00	0.29	0.50
570	Random Forest	29	40	19	8	21	0.52	0.72	0.61	0.67
575	MLP	46	53	17	10	36	0.68	0.78	0.73	0.76
584	Decision Tree	15	20	11	6	9	0.45	0.60	0.51	0.56
588	AdaBoost	43	49	17	11	32	0.65	0.74	0.70	0.72
591	AdaBoost	40	65	35	10	30	0.46	0.75	0.57	0.67
596	Gradient Boosting	44	54	25	15	29	0.54	0.66	0.59	0.63

*FP* false positives, *FN* false negatives, *TP* true positives, *PPV* (*precision*) positive predictive value, *TPR* (*recall*) true positive rate

Table 4 displays a summary of the evaluation results for a single participant (588). Each classifier has a specific number of predicted meals, and this impacts the number of false alarms (**FP**), as well as detected meals (**TP**). When looking at aggregated results such as this, it is difficult to realize how harmful the number of false alarms are, or how good the detection is. For that, in Table 5 the results of the application of the method using the selected classifier for this participant (AdaBoost) is shown, but now with results for each of the tested days.

**Table 4:** Model Selection metrics. Resulting values of the model selection metrics of the models validated on participant 588 data sorted by *F*$_\beta$*-score*

ID:588 Meals: 43								
Classifier	Predictions	FP	FN	TP	PPV	TPR	F$_1$-score	F$_2$-score
AdaBoost	49	17	11	32	0.65	0.74	0.70	0.72
Random Forest	41	12	14	29	0.71	0.67	0.69	0.68
Gradient Boosting	47	17	13	30	0.64	0.70	0.67	0.68
Decision Tree	38	13	18	25	0.66	0.58	0.62	0.60
MLP	38	13	18	25	0.66	0.58	0.62	0.60
Gaussian Naive Bayes	36	15	22	21	0.58	0.49	0.53	0.50

*FP* false positives, *FN* false negatives, *TP* true positives, *PPV* (*precision*) positive predictive value, *TPR* (*recall*) true positive rate

**Table 5:** Validation results per day for participant 588. Resulting values per day of the selected classifier (AdaBoost) validation on participant 588 data

ID:588 Classifier: AdaBoost									
Day	Meals	Predictions	FP	FN	TP	PPV	TPR	F$_1$-score	F$_2$-score
2021-10-05	4	6	2	0	4	0.67	1.00	0.80	0.91
2021-10-06	4	6	2	0	4	0.67	1.00	0.80	0.91
2021-10-07	4	5	2	1	3	0.60	0.75	0.67	0.71
2021-10-08	5	4	1	2	3	0.75	0.60	0.67	0.62
2021-10-09	3	5	3	1	2	0.40	0.67	0.50	0.59
2021-10-10	3	2	1	2	1	0.50	0.33	0.40	0.36
2021-10-11	5	6	2	1	4	0.67	0.80	0.73	0.77
2021-10-12	6	4	2	4	2	0.50	0.33	0.40	0.36
2021-10-13	4	6	2	0	4	0.67	1.00	0.80	0.91
2021-10-14	5	5	0	0	5	1.00	1.00	1.00	1.00
Average	4.3	4.9	1.7	1.1	3.2	0.64	0.75	0.68	0.71

*FP* false positives, *FN* false negatives, *TP* true positives, *PPV* (*precision*) positive predictive value, *TPR* (*recall*) true positive rate

The average number of **FP** is 1.7, meaning that if in a scenario where a daily tracking application makes use of the method, the participant would receive around one or two notifications/reminders that would be ignored. For the **FN**, the average is 1.1, hence, for each day, a single meal would not be detected. On the other hand, the user would be notified correctly regarding 3 detected meals (**TP**) per day in average, being able to “forget” such meals as the system would remind him/her.

### Testing: full selection and classification

For a more illustrative explanation on how the selected candidates are classified, all steps regarding such procedure will be given following the results of the same previously used participant (588), however now covering the method applied to the *testing* set, and Table 6 presents the first of the associated results.

**Table 6:** Test results per day for participant 588. Resulting values of the selected model (AdaBoost) tested on participant 588 data

ID:588 Classifier: AdaBoost									
Day	Meals	Predictions	FP	FN	TP	PPV	TPR	F$_1$-score	F$_2$-score
2021-10-15	5	4	3	4	1	0.25	0.20	0.22	0.21
2021-10-16	2	3	2	1	1	0.33	0.50	0.40	0.45
2021-10-17	4	8	6	2	2	0.25	0.50	0.33	0.42
2021-10-18	3	2	1	2	1	0.50	0.33	0.40	0.36
2021-10-19	4	7	3	0	4	0.57	1.00	0.73	0.87
2021-10-20	4	6	2	0	4	0.67	1.00	0.80	0.91
2021-10-21	4	4	0	0	4	1.00	1.00	1.00	1.00
2021-10-22	2	5	3	0	2	0.40	1.00	0.57	0.77
2021-10-23	2	3	1	0	2	0.67	1.00	0.80	0.91
2021-10-24	4	8	4	0	4	0.50	1.00	0.67	0.83
Average	3.4	5.0	2.5	0.9	2.5	0.51	0.75	0.59	0.67

*FP* false positives, *FN* false negatives, *TP* true positives, *PPV* (*precision*) positive predictive value, *TPR* (*recall*) true positive rate

**Fig. 3:**
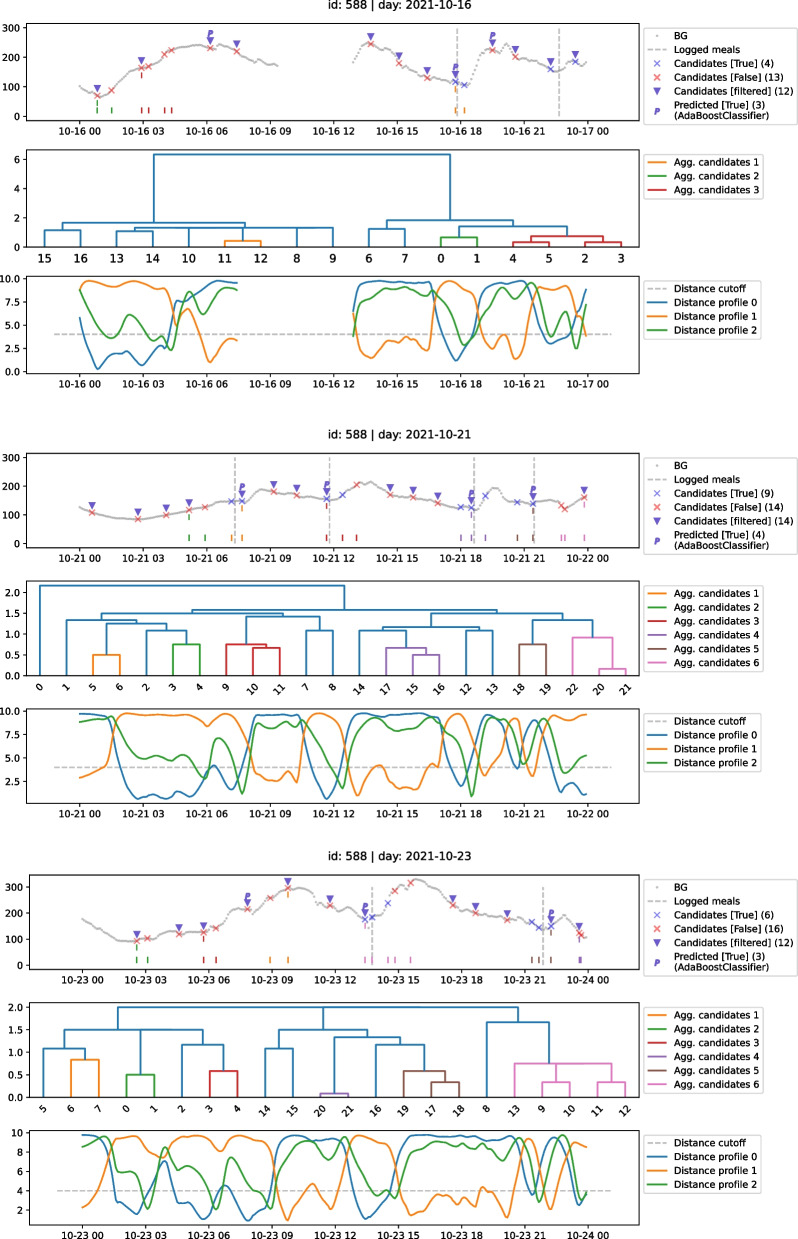
Classification data. Classified candidates used as predicted/detected meal flags for participant 588 events on days 2021-10-16, 2021-10-21, and 2021-10-23

Figure 3, which has sample days from the *testing* set of the same previously used participant (588), can be used to describe the full procedure of selecting and classifying the candidates through an illustrative explanation. The classification proceeds in the same way for each day, and thus it can be followed through any of the depicted samples.

For every sample day, the top plot contains the BG signal together with the reported meals, where the latter is marked by vertical dashed lines colored in gray. The distance profiles are plotted in the bottom, and as already mentioned, each associated to one of the used patterns *P*. In addition, the distance cutoff value used ($d_{cutoff}=4$) is depicted as a horizontal gray line.

Using $\Delta _{valley}=2$ hour, the full resulting set of selected candidates *C* are marked by crosses: blue indicating candidates that are within the success margin (for this example, must be seen as meals if within 1 hour from the logged meal event), and red for the ones outside. One can note that the valleys shown in the plotted distance profiles below the $d_{cutoff}$ threshold are in-sync with the marked candidates, which is a very important aspect of the selection. The filtered version of the candidates *C*, resulting from the agglomerative clustering step (see Algorithm 2, Additional file 1), are marked by triangle shaped markers. The elements of each agglomeration made by the dendogram presented in the middle of the figure are marked right above the *x* axis of the bottom plot by vertical bars. Each bar color is the same associated to the agglomerations. During this step, the number of selected candidates is reduced significantly, for instance, by 10 on day 2021-10-21, going from 24 to 14, i.e., 10 elements of *C* were ignored because they are close enough to other candidate(s) more similar to the pattern.

With every – now filtered – selected candidate at hand, the classification takes place. From the trained classifiers, the estimator taken as the general best for the participant is used, this being the result of the evaluation done using the participant’s *validation* set during the model selection. *AdaBoost* was the model of choice for participant 588, and thus all positive predictions made by this classifier for the filtered version of the candidates *C* are marked with a capital P.

In Fig. 3, each sample day has a different number of logged meals *E*, and the objective is to match them with the predicted P’s. The plots show that a meal was correctly predicted (true positive) when a P marks a blue cross, a false alarm (false positive) happens when a P marks a red cross, and an overlooked meal event (false negative) when no P marks any blue cross around a logged meal. For instance, in the first day plotted, 2021-10-16, only 1 of the meals was correctly predicted, with 2 false alarms, and 1 overlooked meal event. On day 2021-10-21, 4 out of 4 meals were matched by the predicted, and no false flags happened, meaning a perfect prediction. On the other hand, for 2021-10-23, 2 out of 2 meals were predicted correctly, however a false flag happened between the marks of 06:00 and 09:00.

Let us take the false flag around the mark of 07:00 for both 2021-10-16 and 2021-10-23. Considering its timestamp and how similar the candidates are to the pattern (valleys in their associated distance profiles), this false flags can be associated with meal events not reported, which would mean that the participant indeed had the meals, however they were not logged by him/her. Also, the fact that the same participant has logged a meal around the same timestamp on 2021-10-21 – as well in other days –, enforces such assumption. Thus, what is now being seen as a prediction mistake from the model’s perspective, could be seen as an on-point inference for data quality improvement, or even a reminder for a log entry not made by a participant.

The displayed example emphasizes the possibility of applying our method to detect meals in order to improve gathered data quality. Another important aspect is the level of personalization of the model: all the detection steps are made using data coming from one participant (588), meaning that the analysis and models created are data-driven and personalized (from individual patterns identified per participant).

Table 7 displays the results of the method application on the data of each of the participants. The model results per participant are displayed in each row, also highlighting the classifier that achieved the best performance. Such results are depicted to show that in the classification step of the pipeline, multiple types and/or instances of classifiers can be used, allowing for model selection to be applied, this way providing room to dynamically define which classifier fits better each of the participants (data). It is worth noting that, for the used set of classifiers, the classification task does not perform well for all the participants. Restricting the attention to participants with F$_2$-score lower than 0.5, it is possible to note that the number of meals reported are lower in the *testing* set than in their own *training* and *validation* sets. The proportion of meals distributed in the dataset plays an important role on the quality of the results achieved. It is not clear from this study if such fact happens due to a poor reporting in the later days of the data gathering – and the model is detecting meal events that were supposed to be there, and are taken into account as mistakes –, or if there is a behavior change from the participant and the model was not able to cope with it.

**Table 7:** Test results for all participants^a^. Resulting values of the selected model tested on the data of each of the participants

ID	Classifier	Meals	Predictions	FP	FN	TP	PPV	TPR	F$_1$-score	F$_2$-score
540	Decision Tree	20	9	8	19	1	0.11	0.05	0.07	0.06
544	Random Forest	32	38	9	3	29	0.76	0.91	0.83	0.87
552	MLP	14	18	12	8	6	0.33	0.43	0.38	0.41
559	Gaussian NB	23	31	20	12	11	0.35	0.48	0.41	0.45
563	Decision Tree	23	28	18	13	10	0.36	0.43	0.39	0.42
570	Random Forest	31	37	13	7	24	0.65	0.77	0.71	0.75
575	MLP	37	42	18	13	24	0.57	0.65	0.61	0.63
584	Decision Tree	20	34	23	9	11	0.32	0.55	0.41	0.48
588	AdaBoost	34	50	25	9	25	0.5	0.74	0.6	0.67
591	AdaBoost	37	64	38	11	26	0.41	0.7	0.51	0.61
596	Gradient Boosting	45	59	27	13	32	0.54	0.71	0.62	0.67

*FP* false positives, *FN* false negatives, *TP* true positives, *PPV* (*precision*) positive predictive value, *TPR* (*recall*) true positive rate

^a^Patient 567 was not included due to the lack of logged meal events

## Conclusions

The work presented in this paper explores a data-driven method of selecting and classifying segments of a glucose signal (CGM) as responses to meal events self-reported by people with diabetes. In the proposed method, the BG signal is translated into a sequence of candidates, where each of them is formed by a set of qualitative variables associated to their contained response shape. This is a form of qualitative representation of the entire BG signal, i.e., a translation of the data into features that contain the needed amount of information.

Existing diabetes management systems could incorporate the proposed detection method to aid users in their daily events routines. By identifying patterns and detecting specific BG variations associated to events, the system could extract the features from the data, classify events that happened, and provide room for specifically signalizing, notifying, and/or nudging towards a better condition management and care. While applicable to meal detection and quality improvement of data consisting of nutritional self-reports, the novel methods of this paper also pave the way to be used for detection and improvement of other events and related data. This aspect also allows for the use of multiple signals together, e.g., originated by different sensors such as photoplethysmogram (PPG) or galvanic skin response. That even expands it to a multi-variate approach, while still making use of the same pipeline now composing different in-parallel extracted features.

The novel procedure for selection of candidates proved to suit well the dynamics of the problem: patterns are associated to different response shapes, hence, the number of times they occur can – and probably will – also differ. Assigning a static value to the number of occurrences (pattern matches) is a non-optimized and arbitrary choice that works well as a starting point for model specification, however does not suit well for more dynamic and complex scenarios. The dynamic selection approach then gives the opportunity for patterns with more matching occurrences to mark more candidates, as (i) only matches with high values of similarity will stay after applying the distance cutoff, and (ii) the *GetValleys* procedure will return only candidates closer to the lower points. This resulted in a lower amount of matches, while maximizing their similarity to the patterns.

The approach – as a modular pipeline – provided the possibility to use different classifiers for the classification step. The goal was to show that any binary classifier that fits better the data/problem faced can be used, and for that no optimization was performed nor was it investigated which type of classifier would fit better in a general manner, and why. For that, as a future work, a more specialized study to analyze the performance of classifiers in detail is intended to be performed.

The uncertainty in the data makes the problem tackled fit well the “chicken or the egg” causality dilemma. The approach tries to improve data quality by detecting meals, and a possible application of such detection is to remind users of missing or wrongly informed meal events. However, the models are trained on real data (not synthetically generated through simulators [19, 49]), which are very likely to contain the same type of issues: potentially wrong reported and missing values. This means that ideally, the first data batch used for modeling must have a certain level of reliability in order to generate the models. Although preferable, and somehow limiting, this point was not taken as a requirement, and the method could still handle such data uncertainty when generating fitting models. This also opens the opportunity to first create more general models from better quality data, and then use such models as base for others. In this way, the base models would be more reliable, and could be evolved with new incoming data to reach an individualized version of it. In addition, although individual models were the scope of choice, a comparison between modeling in population and individual levels in the same fashion as the one done in [15] is a future step to be taken in the research, including specific analysis over how much data is necessary to train the models while maintaining accuracy.

### Supplementary Information

**Additional file 1.**

## Data Availability

The dataset (OhioT1DM), analyzed during this study, is properly cited, and publicly available from the corresponding author [28] on request at the link https://ohio.qualtrics.com/jfe/form/SV_02QtWEVm7ARIKIl. All code base regarding the experiments and algorithms described by the authors can be available upon direct request.

## Footnotes

1 Patterns are found using the *training* subset only.

2 https://github.com/scikit-learn/scikit-learn

3 https://github.com/TDAmeritrade/stumpy

4 https://smarthealth.cs.ohio.edu/OhioT1DM-dataset.html
