# Supplementary material for: Data-driven meal events detection using blood glucose response patterns
Source: BMC Med Inform Decis Mak. 2023 Dec 8;23:282. doi: 10.1186/s12911-023-02380-4 (PMC10709931; doi:10.1186/s12911-023-02380-4)
Supplement: Supplementary file 1 — Additional file 1. [file 12911_2023_2380_MOESM1_ESM.pdf]

SUPPLEMENTARY MATERIAL

# Details on Response Patterns and Candidates Selection Methods

Danilo F. de Carvalho[1*], Uzay Kaymak[1], Pieter Van Gorp[2] and Natal van Riel[3]

**Primary manuscript:** Data-driven Meal Events Detection using Blood Glucose Response Patterns

*Correspondence:
d.ferreira.de.carvalho@tue.nl
[1]Jheronimus Academy of Data Science, Eindhoven University of Technology, 's-Hertogenbosch, The Netherlands
[2]Industrial Engineering & Innovation Sciences, Eindhoven University of Technology, Eindhoven, The Netherlands
[3]Department of Biomedical Engineering, Eindhoven University of Technology, Eindhoven, The Netherlands
Full list of author information is available at the end of the article

## Introduction

This additional file is dedicated to give supplementary content to the methods described in our work with title "Data-driven Meal Events Detection using Blood Glucose Response Patterns". It must be taken as a direct support content to the latter, giving extra information and serving as an overview of specific concepts and steps, facilitating the understanding of important aspects of the work presented in the primary manuscript.

## Identifying Response Patterns

The responses gathered from events are pieces of the blood glucose (BG) time series with specific characteristics, and so can be seen as *shapelets* [1, 2], *i.e.*, a representation of a class that carry enough information to allow their identification within a time series. Each response has its own uniqueness, however they tend to share properties implicit to their shapes, and so can be grouped according to these common facets [3]. The premise is that each resulting group/cluster can represent their members while keeping their core information, and so preserve closely the original aspects of their members' data [4]. Previous works have shown methods on grouping time series by their similarities making use of clustering algorithms [5, 6, 7, 8, 9, 10], and as part of our methods, we used the patterns' identification step described in our previous work [11], which follows the same rules and aspects. For our approach, the center of each meal response cluster formed is used as a pattern, hence the number of identified patterns is equal to the number of clusters used during this same step.

## Looking for Matches

After having the response patterns defined, and thus a set of patterns $P = \{p_0, p_1, \ldots, p_i\}$, our proposed approach relies on searching for occurrences of such patterns in the BG time series $T$ by shape similarity. For that, the MASS [12] algorithm is used, which generates and returns one distance profile [1] per queried pattern.

**Definition 1** (Distance Profile) Let $Q$ be a subsequence taken as the query. For all the possible windows of the same length of $Q$ obtained by sliding across a time series $T$, the Euclidean distance to $Q$ is calculated and stored in a vector $d$. Such vector is called *distance profile* in regard to the query $Q$, and have cardinality $|T| - |Q| + 1$.

The set $D = \{d_1, d_2, \ldots, d_i\}$, which includes all distance profiles generated using patterns $P = \{p_0, p_1, \ldots, p_i\}$ as queries, is generated over the entire BG signal taken as a time series $T$, and become a fundamental part of the matches selection [1].

Algorithm 1 generalizes the match selection procedure applied in [11].

**Algorithm 1** Selecting Matches

```
Require: T, D, n                          ▷ n is the number of top matches per pattern
Ensure: Matches as a subset of D
1: procedure SelectMatches(T, D, n)
2:     C ← 2-D array of shape (len(D), n)
3:     for i = 0, 1, ..., len(D) do                       ▷ D = {d_0, d_1, ... d_i}
4:         d ← D[i]                        ▷ Distance profile associated to pattern i
5:         C[i] ← SelectTopMatches(d, n)
6:     end for
7: end procedure
```

Some important points are worth highlighting regarding this procedure:

- Patterns $P$ are not needed in this step, as $D$ contains the distance profiles generated with – and associated to – each of them.
- The procedure works right away for the entire BG time series $T$, or any subsequence of it, as the associated distance profiles are already known.
- $SelectTopMatches$ (line 5 of Algorithm 1) returns the $n$ sorted indices by distance, resulting in a per pattern top-$n$ smallest distance (*N.B.* highest similarity) approach.
- The number of matches created is $|C| = n \times |P|$.

In order to make the mentioned points clear, a sample of the applied procedure can be shown in Figure 1. Matches are selected for a sample day of participant 588, in other words, a subsequence of $T$ (to be more precise, $T_{588}$), using three patterns, and $n = 10$. On the left side, each queried pattern is plotted together with the subsequence with the smallest distance to the pattern (also highlighted in the BG plot to the right). On the right side of the same figure, for each pattern, the BG time series and the associated distance profile are plotted, the latter together with triangle shaped marks placed on the starting point of each of the top-10 smallest matches. The meal events are also present, and marked by vertical dashed lines.

One can note that all patterns had matches grouped in valleys of their respective distance profiles. These valleys determine a group of neighbor subsequences that have similar shape to the pattern, thus it is expected that for a match associated to the lowest point (bottom) of a valley, a number of neighbor subsequences will be comparable to it but less similar to the pattern, explaining the formed groups, and making them the relevant match marking spots.

By analyzing the distance profiles, and how they vary creating these aforementioned valleys, it is worth noting that by increasing the value of $n$, more matches will be spotted inside valleys the same way. In addition, for each increment in $n$, the amount of matches is incremented by the number of patterns. For the meal detection case, this ends up as a tweaking issue, where the optimum value for $n$ able to return a reasonable number of matches is unknown due to how different the distance profiles generated per pattern are.

[1] Associated, $D$ and $P$ can also be seen as a set of pairs $\{(p_0, d_0), (p_1, d_1), \ldots, (p_i, d_i)\}$.

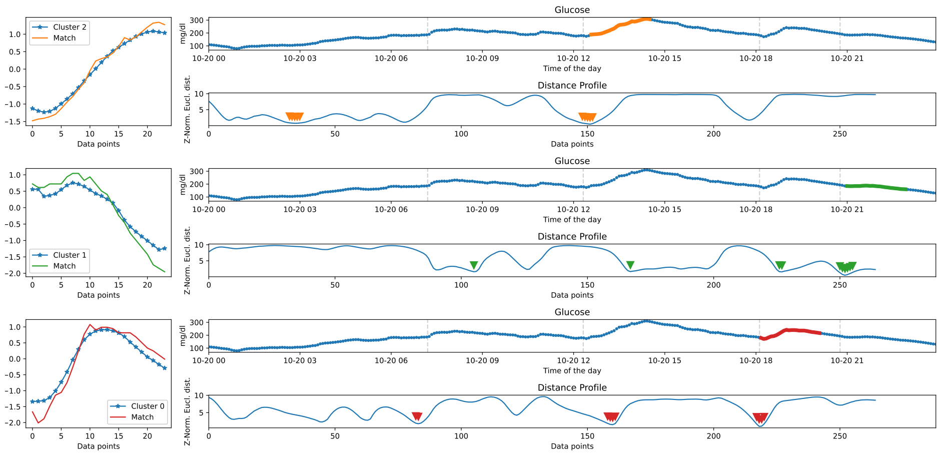


**Figure 1 Sample of identified matches.** Matches found on day 2021-10-20 for participant 588 using the top-10 smallest distance-to-pattern approach [13].

For instance, an approach focusing on detecting three main meals in a day could start by using $n = 1$, allowing each of the patterns to mark a single match, resulting in 3 matches a day. This might lead to a perfect score, only if each pattern detects exactly one type of meal (*e.g.*, breakfast, lunch, and dinner). However, this approach is restricting the patterns to happen only once in a day, which in a practical setting is not what happens. Still in the $n = 1$ scenario, if a pattern is also tied to an event that happen more often in a day (*e.g.*, snacks, hypo-correction), it would still be limited to 1 match, flagging exclusively a main meal or a possible snack. Intuitively, an increment of $n$ can be made, reaching now $n = 2$. This would allow that specific pattern to mark a snack, however it would also force the other patterns to mark an extra occurrence on top of their already marked meals, artificially pushing the number of matches up to 6, very likely leading to errors. This incremental setting tends to be arbitrary and not supportive of exceptional days, which do occur in free living conditions, and are critically important to diabetics.

Depending on the signal, and pattern shapes, the number of occurrences will clearly differ, and one pattern is more likely to have more similar occurrences than others. Thus, determining a dynamic way of having a set of matches for a given set of patterns and input subsequence becomes the next step for a more suitable solution.

## Candidates Selection

From the details given in the previous section, one can note that the selection of matches is a critical part of the proposed method, and that the used top-$n$ approach is not without limitations. With that being said, the procedure of selecting candidates, defined in Algorithm 2 as SelectCandidates, comes as a direct improvement, or at any rate an alternative, to the previous SelectMatches procedure found in Algorithm 1.

The main differences in the proposed candidates approach rely on the use of two new introduced parameters, and how they are used in the algorithm's scope:

**Algorithm 2** Candidates Selection

**Require:** $D, d_{cutoff}, \Delta_{valley}$ ▷ Distance Profiles, distance cutoff, and valley length
**Ensure:** Candidates as a subset of $D$
**procedure** SELECTCANDIDATES($D, d_{cutoff}, \Delta_{valley}$)
  $C \leftarrow$ Empty set of time stamps
  **for** $i = 0, 1, \ldots, len(D)$ **do** ▷ $D = \{d_0, d_1, \ldots d_i\}$
    $d \leftarrow D[i]$ ▷ Distance profile associated to each pattern $i$
    $c \leftarrow ApplyDistanceCutoff(d, d_{cutoff})$
    $c \leftarrow GetValleys(c, \Delta_{valley})$
    $C \leftarrow C + c$ ▷ Add elements from c to C
  **end for**
  $d_{max} \leftarrow \Delta_{valley}/2$ ▷ Max allowed inner cluster distance
  $C \leftarrow AgglomerativeCluster(C, d_{max})$ ▷ Step exclusive to the validation
  **return** $C$
**end procedure**

$d_{cutoff}$

A distance threshold used to filter out subsequences with low similarity to the patterns. On our approach, the value is set by analyzing the estimated distribution of distance values of the associated distance profile. For instance, in Figure 2 such estimation is depicted for participant 588, and in this case, $4 \leq d_{cutoff} \leq 5$ would suffice, as the distance value is low enough to guarantee a good similarity while keeping enough candidates.

$\Delta_{valley}$

Defines how distant the selected candidates must be from each other. Sorted by distance, and moving on an ascending direction – starting from the lowest distance (highest similarity) to the highest (lowest similarity) –, all elements of the generated distance profile are checked. An element is taken, and its neighbors within a $\Delta_{valley}$ margin are ignored, as they are very similar to the element taken as reference, but less similar to the pattern. The next element is taken, and, if not already ignored as a neighbor, the same procedure is applied again. This is done until all elements are checked, and so the remaining are returned as the set of candidates. This must be tweaked according to the type of event in hands: if the events being handled happen with a short period of time between them, $\Delta_{valley}$ must be short enough to allow candidates to appear in a similar frequency.

Figure 3 illustrates the influence of the combined use of $d_{cutoff}$, and $\Delta_{valley}$ during the selection of candidates. Within the highlighted time window, only one of the patterns (Pattern 1, or $p_1$) provide relevant distances, as its distance profile (Distance profile 1, or $d_1$) is the only one that has satisfactory similar occurrences, or values below the distance threshold, $d_{cutoff}$, marked by the gray dashed horizontal line. This distance values are now processed by $GetValleys$, which makes use of the $\Delta_{valley}$ parameter explained earlier, and ends up returning the selected candidates marked by black dots.

Some relevant aspects of the candidates selection can be listed as follows:

- Both new parameters $d_{cutoff}$ and $\Delta_{valley}$ allow for a more flexible control over the number and quality of returned candidates.
  - $d_{cutoff}$ allows for filtering low similarity values straight away.
  - $\Delta_{valley}$ can be specified according to the domain and type of events being handled, and the frequency they commonly happen.

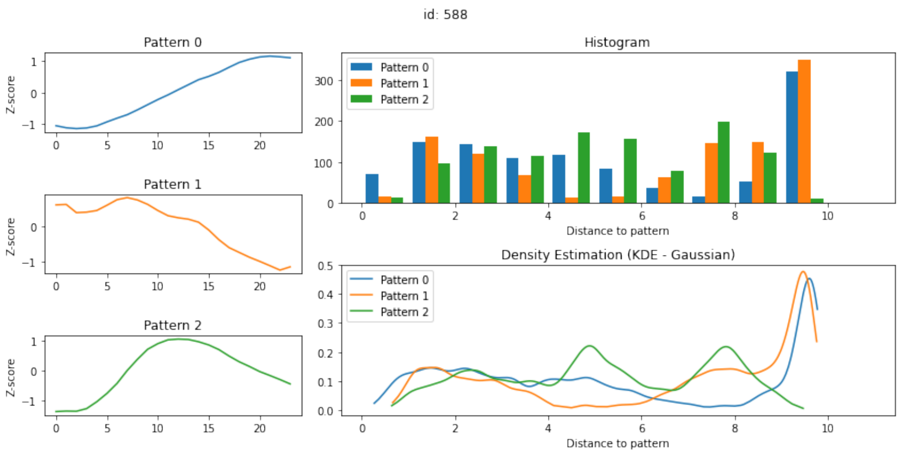


**Figure 2 KDE of the distance profiles for setting $d_{cutoff}$ value.** Kernel Density Estimation (KDE) of the distance profiles used to determine the $d_{cutoff}$ parameter value for participant 588.

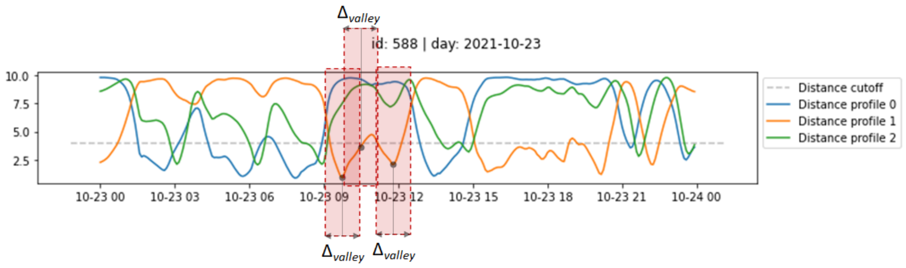


**Figure 3 $GetValleys$ procedure.** Combined use of $d_{cutoff}$, and $\Delta_{valley}$ when selecting candidates in a specific example window of day 2021-10-23 of partcicipant 588, resulting in 3 candidates.

- No pattern is limited or forced to mark a fixed number of candidates, as the most relevant occurrences will be dynamically selected (due to the combined use of $d_{cutoff}$ and $\Delta_{valley}$).

The $GetValleys$ procedure is applied to each distance profile individually, thus the resulting candidates set can still have elements placed too close (or even overlapping) in time. To solve such issue, as a last filtering step, agglomerative clustering [14, 15, 16] is applied on the time axis for the full candidates set. This is done to group candidates that appear too close in a specified period of time, and is controlled by the inner cluster distance $d_{max}$, that in our case is set equal to $\Delta_{valley}/2$. This way we try to keep the same candidates spacing determined by $\Delta_{valley}$ in the previous step, however now for the full resulting candidates set [2].

At this point, the patterns were extensively used as a full data-driven and unsupervised filtering step in order to spot potential meal events through the selected pattern matches/occurrences. With the set of matches at hand, a classification (supervised) step can be done. This is why the method was named after candidates:

[2]During training, this step is skipped, as ignoring candidates could lead to an undesired reduction in the amount of positives to train with.

every similar occurrence of the pattern must not be seen as meals yet, they are now candidates still to be classified as such.

**Author details**
[1]Jheronimus Academy of Data Science, Eindhoven University of Technology, 's-Hertogenbosch, The Netherlands.
[2]Industrial Engineering & Innovation Sciences, Eindhoven University of Technology, Eindhoven, The Netherlands.
[3]Department of Biomedical Engineering, Eindhoven University of Technology, Eindhoven, The Netherlands.

**References**

1. Ye L, Keogh E. Time Series shapelets: A new primitive for data mining. In: Proceedings of the 15th ACM SIGKDD International Conference on Knowledge Discovery and Data Mining. KDD '09. New York, NY, USA: Association for Computing Machinery; 2009. p. 947–956.
2. Yeh CCM, Zhu Y, Ulanova L, Begum N, Ding Y, Dau HA, et al. Time series joins, motifs, discords and shapelets: a unifying view that exploits the matrix profile. Data Mining and Knowledge Discovery. 2018;32(1):83–123.
3. Caiado J, Ann Maharaj E, D'Urso P. Time-series clustering. Handbook of Cluster Analysis. 2015;p. 241–264.
4. Vlachos M, Lin J, Keogh E, Gunopulos D. A Wavelet-Based Anytime Algorithm for K-Means Clustering of Time Series. In: In Workshop on Clustering High Dimensionality Data and Its Applications, at the 3 rd SIAM Int'l Conference on Data Mining; 2003. p. 1–3.
5. Bezdek JC, Ehrlich R, Full W. FCM: The fuzzy c-means clustering algorithm. Computers and Geosciences. 1984 jan;10(2-3):191–203.
6. Liao Warren T. Clustering of time series data – A survey. Pattern Recognition. 2005;38(11):1857–1874.
7. Esling P, Agon C. Time-series data mining. ACM Computing Surveys. 2012 nov;45(1):1–34.
8. Ali M, Alqahtani A, Jones MW, Xie X. Clustering and Classification for Time Series Data in Visual Analytics: A Survey. IEEE Access. 2019;7:181314–181338.
9. Ergüner Özkoç E. Clustering of Time-Series Data. In: Data Mining - Methods, Applications and Systems [Working Title]. IntechOpen; 2020. .
10. Javed A, Lee BS, Rizzo DM. A benchmark study on time series clustering. Machine Learning with Applications. 2020 sep;1:100001.
11. F de Carvalho D, Kaymak U, Van Gorp P, van Riel N. Population and Individual Level Meal Response Patterns in Continuous Glucose Data. In: Information Processing and Management of Uncertainty in Knowledge-Based Systems. IPMU 2022. vol. 1602 Communications in Computer and Information Science. Springer International Publishing; 2022. p. 235–247.
12. Yeh CCM, Zhu Y, Ulanova L, Begum N, Ding Y, Dau HA, et al. Matrix profile I: All pairs similarity joins for time series: A unifying view that includes motifs, discords and shapelets. In: 2016 IEEE 16th International Conference on Data Mining (ICDM); 2016. p. 1317–1322.
13. F de Carvalho D, Kaymak U, Van Gorp P, van Riel N. A Markov model for inferring event types on diabetes patients data. Healthcare Analytics. 2022;2:100024.
14. Chidananda Gowda K, Krishna G. Agglomerative clustering using the concept of mutual nearest neighbourhood. Pattern Recognition. 1978 jan;10(2):105–112.
15. Tokuda EK, Comin CH, Costa LdF. Revisiting agglomerative clustering. Physica A: Statistical Mechanics and its Applications. 2022 jan;585:126433.
16. Cai J, Hao J, Yang H, Zhao X, Yang Y. A review on semi-supervised clustering. Information Sciences. 2023 jun;632:164–200.
